# Possible association of CCDC62 rs12817488 polymorphism and Parkinson’s disease risk in Chinese population: a meta-analysis

**DOI:** 10.1038/srep23991

**Published:** 2016-04-01

**Authors:** Yanjun Lu, Lu Tan, Na Shen, Jing Peng, Chunyu Wang, Yaowu Zhu, Xiong Wang

**Affiliations:** 1 Department of Laboratory Medicine, Tongji Hospital, Tongji Medical College, Huazhong University of Science and Technology, Wuhan 430030, China; 2 Key Laboratory for Molecular Diagnosis of Hubei Province, The Central Hospital of Wuhan, Tongji Medical College, Huazhong University of Science and Technology, Wuhan, Hubei 430014, China

## Abstract

Conflicting results identifying the association between coiled-coil domain containing 62 (CCDC62) polymorphism, rs12817488, and Parkinson’s disease (PD) have been reported. To clarify whether rs12817488 is related to PD risk in Chinese population, we carried out this meta-analysis by searching literature from PubMed and Embase database regarding this polymorphism. Three eligible studies involving 1616 cases and 1649 controls were included in this meta-analysis. Our results showed statistically significant association between rs12817488 and PD risk in all four genetic models. Stratification by gender revealed similar results in both subgroup in these genetic models except for recessive model, in which rs12817488 was not significantly associated with PD in male subgroup. Unstable result was found in recessive model via sensitivity analysis, and publication bias was observed in recessive model as well, indicating that the pooled result from recessive model should be cautiously treated. Our meta-analysis implicates a possible relationship between rs12817488 and PD risk in Chinese population. Further validation of this association in large sample size study with different gender is warranted.

Parkinson’s disease (PD) is the second most frequent neurodegenerative disease, clinically characterized with muscle rigidity, tremors, postural instability, bradykinesia and psychiatric symptoms, and costs on patients, caregivers, and society. The etiology of PD involves both genetic and environmental factors. Fewer than 5% of overall PD cases are attributed to genetic mutations mainly in α-synuclein^1^,^2^,^3^. Therefore, understanding the genetic architecture of sporadic PD cases will be helpful for PD risk prediction and gene therapy.

A number of genes associated with PD risk have been found in both familial and sporadic Patients via genome-wide association studies (GWAS) and high throughput genotyping methods like next generation sequencing, including SNCA, LRRK2, PINK1, SLC45A3, ACMSD, HLA, GBA, RIT2, and CCDC62^4^,^5^. Rs12817488 is localized in the intron of Coiled-coil domain containing 62 (CCDC62), and has been found to be associated with PD risk, but the overall and stratified subgroup results were controversial. Yu RL showed significant association only existed in male, while Liu RR found this only in female population^6^,^7^. However, Li NN found no relationship between rs12817488 and PD susceptibility^8^. Although the three studies invested Chinese population, the results were not consistent.

To clarify whether rs12817488 is related to PD risk in Chinese population, we performed this meta-analysis, aiming to identify the contribution of rs12817488 to PD pathogenesis and to illustrate possible reasons for these conflicting results.

## Methods

### Literature search

Eligible studies were systematically searched in PubMed and Embase databases up to Oct 30, 2015, with keywords including “Parkinson’s disease or PD” and “CCDC62 OR coiled-coil domain containing 62 OR rs12817488” and “polymorphism or mutation or variation or SNP”. We also manually examined reference lists for other relevant publications.

### Inclusion and exclusion criteria

Studies were chosen if they met the following criteria: (1) evaluating association between rs12817488 and PD in Chinese population; (2) a case-control study; (3) available phenotype and allele frequencies data. Reviews, abstracts from conferences, republished or duplicate studies, studies with insufficient information for data extraction were excluded.

### Data extraction

The following information was collected: (1) first author and publication year; (2) country and ethnicity; (3) sample size and sex ration; (4) phenotype distribution and minor allele frequency (MAF).

### Statistical analysis

We used STATA software 11.0 (STATA Corp., College Station, TX, USA) for all statistical analyses. The departure of rs12817488 frequencies from expectation under Hardy-Weinberg equilibrium (HWE) was assessed by chi-square in control group, and it was considered to be disequilibrium if *P* < 0.05. The pooled odds ratios (OR) and 95% confidence intervals (CI) were calculated by the Z test to evaluate this association under the allelic (A vs. G), dominant (AA + GA vs. GG), recessive (AA vs. GA + GG), and additive (AA vs. GG) genetic models. Heterogeneity among studies was tested using Q test and I^2^ statistic. If *P*_Q_ > 0.10 or I^2^ < 50%, the pooled OR was estimated by fixed-effect model. Otherwise, random-effect model was applied. Sensitivity analysis was conducted by sequentially excluding each study to assess the stability of the results. Publication bias was assessed by Begg’s and Egger’s tests. *P* < 0.05 was considered significant for all tests.

## Results

### Characteristics of published studies

A total of 36 studies were retrieved (PubMed: 18, Embase: 18). As the result in Embase was the same with that in PubMed, therefore, 18 studies were retrieved. 1 review and 14 irrelevant studies were excluded. Finally, 3 eligible studies (1616 cases and 1649 controls) published from 2013 to 2015 were chosen, and the data were extracted^6^,^7^,^8^. The genotype frequencies of rs12817488 in controls of each study met the HWE expectation (*P* > 0.05). The genotype distributions of all studies are summarized in Tables 1, 2, 3.

### Meta-analysis of rs12817488

**Figure 1:**
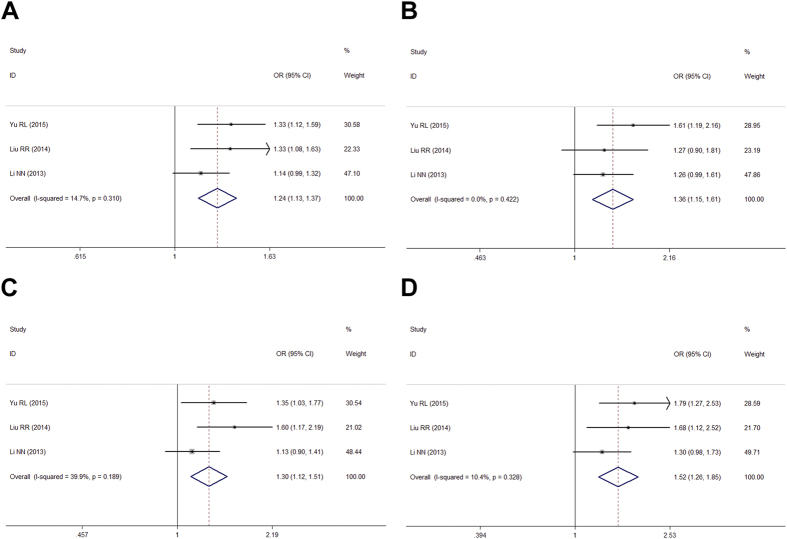
Forest plots for meta-analysis of rs12817488 polymorphism and PD risk in overall Chinese population. (**A**) Allelic model (A vs. G). (**B**) Dominant genetic model (AA + GA vs. GG). (**C**) Recessive genetic model (AA vs. GA + GG). (**D**) Addictive genetic model (AA vs. GA).

Overall, heterogeneity in the four genetic models was not statistically significant, and the ORs and 95% CIs were therefore calculated in fixed-effect model (Table 4). Pooled ORs showed that rs12817488 was significantly associated with an increased PD risk in all four genetic models (allelic, dominant, recessive, and additive models) in Chinese populations (Table 4, Fig. 1).

**Figure 2:**
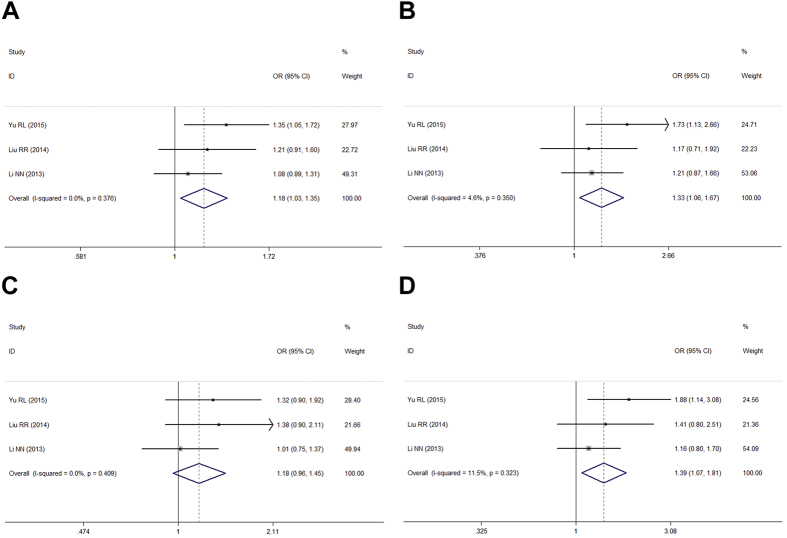
Forest plots for meta-analysis of rs12817488 polymorphism and PD risk in male Chinese population. (**A**) Allelic model (A vs. G). (**B**) Dominant genetic model (AA + GA vs. GG). (**C**) Recessive genetic model (AA vs. GA + GG). (**D**) Addictive genetic model (AA vs. GA).

**Figure 3:**
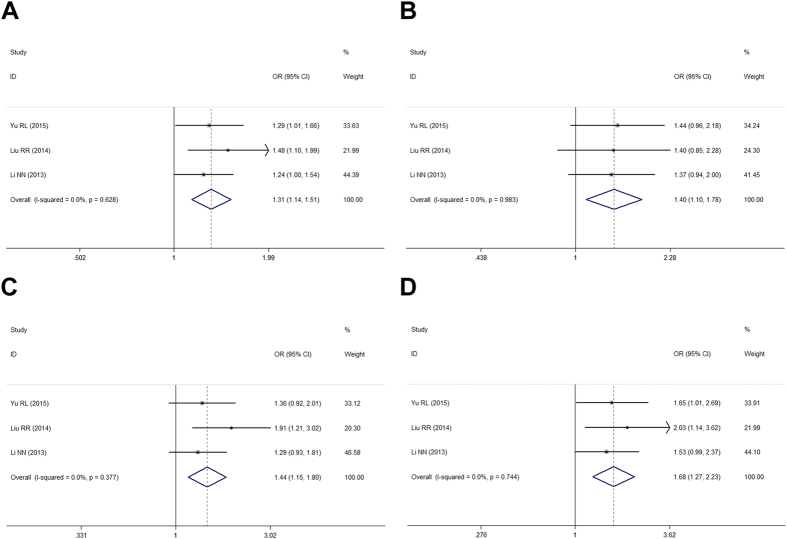
Forest plots for meta-analysis of rs12817488 polymorphism and PD risk in female Chinese population. (**A**) Allelic model (A vs. G). (**B**) Dominant genetic model (AA + GA vs. GG). (**C**) Recessive genetic model (AA vs. GA + GG). (**D**) Addictive genetic model (AA vs. GA).

Stratification was performed by gender (male vs. female), and significant association was found in all genetic models within the female subgroup (Table 4, Fig. 2). In the male subgroup, rs12817488 was remarkably associated with PD in allelic, dominant, and additive models, while in recessive model, rs12817488 was not significantly associated with PD risk (OR = 1.18, 95% CI:0.96, 1.45, *P* = 0.119; Table 4, Fig. 3).

### Sensitivity analysis

The sensitivity analysis showed that after omitting Liu RR or Li NN’s study, rs12817488 was associated with PD (OR = 1.22, 95% CI:1.02, 1.45, *P* = 0.027; OR = 1.46, 95% CI:1.19, 1.79, *P* = 0.000, respectively) in recessive model. In allelic, dominant, and additive models, the results were stable (Table 5). These data indicate that the pooled results remain robust in allelic, dominant, and additive models but were unstable under recessive model in Chinese population.

### Publication bias

Potential publication bias in this meta-analysis was examined by Begg’s and Egger’s tests. A publication bias was found in recessive genetic model, and no publication bias was detected in allelic, dominant, or additive model (Table 6). The results showed no evidence of obvious asymmetry for most genetic models, while insufficient for recessive model.

## Discussion

Rs12817488 is located in the intron of CCDC62 transcript variant 2 within 12q24.31. CCDC62 is involved a variety of biological processes, including cell growth, cyclin D1 expression, and estrogen receptor activation, and antibodies to CCDC62 develop in various malignancies^9^,^10^. Up to now, functional studies of CCDC62 remain poor, and are mainly associated with cancer. A large scale meta-analysis of GWAS in PD identified 5 novel PD genetic loci (SYT11, ACMSD, MCCC1/LAMP3, STK39, and CCDC62), and since then, studies of CCDC62 in PD increased slowly^11^.

Li NN found that rs12817488 did not associate with PD risk in either male or female population^8^. Liu RR showed that rs12817488 A allele was significantly associated with elevated PD risk, and this association only existed in females. They also detected the protein level of CCDC62 in peripheral blood mononuclear cells from 41 AA or GG carriers and they found that CCDC62 level in PD patients carrying the AA genotype was apparently higher than GG carrier, suggesting that this locus of CCDC62 might be functional^6^. Yu RL showed that rs12817488 was associated with PD risk in late-onset PD (LOPD) patients and controls, but not the early-onset PD (EOPD) patients and controls. They found that allele frequencies and genotype between male PD patients and male controls were significantly different, while there was no difference in female^7^. Collectively, these data suggest that CCDC62 may play an important role in PD pathogenesis, but it may act diversely in different gender. Thus, we perform this meta-analysis to investigate the pooled effect size of this association. As all the three studies explored the association between rs12817488 and PD risk in Chinese population, we mainly focused on Chinese population.

Significant association of rs12817488 with PD risk was found in the pooled Chinese population under the four genetic models. Stratification by gender found similar results with the overall population except for recessive genetic model in male population. Sensitivity analysis further showed that the association between rs12817488 and PD risk was stable in allelic, dominant, and additive models, but remained unstable in recessive genetic model. Furthermore, a publication bias existed in recessive model, indicating a limited number of studies for recessive model in the current meta-analysis. Taken together, our meta-analysis stably showed significant association between rs12817488 and PD risk in major genetic models, while the pooled result from recessive model should be cautiously treated due to the instability.

The prevalence of PD ranged from 1% to 3%, and the prevalence of PD in China is about 1.7%^12^,^13^,^14^. In our meta-analysis, 1616 cases and 1649 controls (3232 and 3298 allele numbers respectively) were included, and the OR was 1.24 in allelic genetic model with a control group MAF of 0.508. We calculated the power of the current meta-analysis at two kind free websites^15^,^16^, (http://csg.sph.umich.edu//abecasis/CaTS/ and http://biostat.mc.vanderbilt.edu/wiki/Main/PowerSampleSize). The power were 0.992 and 0.991 respectively (Figure S1), and the needed case and control allele numbers were 3221 and 3285 which were smaller than the sample patient numbers we used, indicating that the sample number used in this study was enough.

Several aspects may contribute to the different results in the 3 studies, including onset age, EOPD/LOPD ratio, sex ratio, MAF. For onset age, the mean onset age in Yu RL’s study was 54.4 ± 12.3, which was 54.19 ± 10.61 in Li NN’s study, while Liu RR did not show the onset age of PD patients. No significant difference appeared here. The definition of EOPD and LOPD was controversial among the 3 studies. Li NN divided into two subgroups according to the age of onset (<50 years of age and ≥50 years of age). In Liu RR’s study, patients with an age at onset <55 years were defined as EOPD, while others were described as LOPD. In Yu RL’s study, the cutoff age was 45 years of age. Li NN *et al.*, showed no association in either EOPD or LOPD subgroup. Yu RL found significant different genotype and allele frequency only in LOPD subgroup, while no difference was found in EOPD subgroup. The controversial results of EOPD/LOPD may be due to the different definition of EOPD/LOPD. Till now, the definition of EOPD/LOPD is not clear^17^,^18^, and we did not perform stratified analysis by EOPD/LOPD. Sex ratio may also contribute to the overall result. In Li NN and Yu RL’s studies, the PD group had a higher male/female ratio, while in Liu RR’s study, the control group had a higher male/female ratio. Yu RL showed significant difference between male PD patients and male control, without difference between female PD patients and female control. Liu RR reported an opposite result with Yu RL. Li NN showed no difference in either male or female population. It should be noted that in male subgroup of Li NN’s study the MAF of control group was 0.035, suggesting that the genotype and allelic frequency in the male control departed from HWE, and it might affect the result. These data suggest that a balanced sex ratio between PD patients and control groups may help avoid the bias, and further studies focusing on different gender and PD risk are also needed.

Some limitations existed in the current meta-analysis that must be considered. First, we only searched literature written in English language. Second, we only analyzed Chinese population. Third, we performed stratification only by gender, without referring other factors. Fourth, study and subject numbers were relatively small, and therefore, our result may be underpowered.

Our meta-analysis suggests a role of rs12817488 in PD pathogenesis in Chinese population. However, future studies with even larger sample size different sex ratio, and different ethnic population should be done to confirm the findings of the current meta-analysis.

## Additional Information

**How to cite this article**: Lu, Y. *et al.* Possible association of CCDC62 rs12817488 polymorphism and Parkinson’s disease risk in Chinese population: a meta-analysis. *Sci. Rep.* **6**, 23991; doi: 10.1038/srep23991 (2016).

## Supplementary Material

Supplementary Information

## Figures and Tables

**Table 1:** Characteristics of 3 studies included in this meta-analysis.

Author	Year	Country	Ethnicity	Sex ratio (M/F)		Sample size	
				Case	Control	Case	Control
Yu RL	2015	China	Asian	1.23	0.84	515	518
Liu RR	2014	China	Asian	1.09	1.15	341	423
Li NN	2013	China	Asian	1.35	1.13	760	708

M: male, F: female.

**Table 2:** Genotype frequencies of rs12817488 in 3 studies included in this meta-analysis.

Author	Case			Control			MAF		HWE
	AA	GA	GG	AA	GA	GG	Case	Control	
Yu RL	164	256	95	133	247	138	0.567	0.495	0.293
Liu RR	121	154	66	108	216	99	0.581	0.511	0.655
Li NN	234	366	160	200	330	178	0.549	0.516	0.075

**Table 3:** Stratified genotype frequencies of rs12817488 in 3 studies included in this meta-analysis.

Author		Case			Control		
		AA	GA	GG	AA	GA	GG
Yu RL	Male	94	145	47	64	112	60
	Female	70	111	48	69	135	78
Liu RR	Male	61	85	32	62	118	46
	Female	60	69	34	46	98	53
Li NN	Male	128	208	100	109	167	99
	Female	106	158	60	91	163	79

**Table 4:** Meta-analysis of rs12817488 polymorphism and risk of PD in Chinese population.

Genetic model	*P*_Q_	I^2^	OR	95% CI	*P*_Z_
Overall					
A vs. G	0.310	14.7%	1.24	1.13, 1.37	<0.001
AA + GA vs. GG	0.422	0.0%	1.36	1.16, 1.61	<0.001
AA vs. GA + GG	0.189	39.9%	1.30	1.12, 1.51	0.001
AA vs. GG	0.328	10.4%	1.52	1.26, 1.85	<0.001
Male					
A vs. G	0.376	0.0%	1.18	1.03, 1.35	0.014
AA + GA vs. GG	0.350	4.6%	1.33	1.06, 1.67	0.015
AA vs. GA + GG	0.409	0.0%	1.18	0.96, 1.45	0.119
AA vs. GG	0.323	11.5%	1.39	1.07, 1.81	0.015
Female					
A vs. G	0.628	0.0%	1.31	1.14, 1.51	<0.001
AA + GA vs. GG	0.983	0.0%	1.40	1.10, 1.78	0.006
AA vs. GA + GG	0.377	0.0%	1.44	1.15, 1.80	0.001
AA vs. GG	0.744	0.0%	1.68	1.27, 2.24	<0.001

**Table 5:** Sensitivity analysis of the meta-analysis.

Genetic model	*P*_Q_	I^2^	OR	95% CI	*P*_Z_
A vs. G					
Yu RL	0.240	27.5%	1.20	1.07, 1.35	0.002
Liu RR	0.177	45.1%	1.22	1.09, 1.36	0.001
Li NN	0.965	0.0%	1.33	1.17, 1.52	<0.001
AA + GA vs. GG					
Yu RL	0.960	0.0%	1.26	1.04, 1.54	0.022
Liu RR	0.214	35.2%	1.39	1.15, 1.68	0.001
Li NN	0.322	0.0%	1.46	1.16, 1.83	0.001
AA vs. GA + GG					
Yu RL	0.074	68.7%	1.32	0.94, 1.86	0.108^a^
Liu RR	0.316	0.4%	1.22	1.02, 1.45	0.027
Li NN	0.418	0.0%	1.46	1.19, 1.79	<0.001
AA vs. GG					
Yu RL	0.312	2.1%	1.42	1.12, 1.79	0.003
Liu RR	0.164	48.5%	1.48	1.19, 1.85	<0.001
Li NN	0.815	0.0%	1.74	1.34, 2.27	<0.001

^a^calculated with random model.

**Table 6:** Publication bias analysis of the meta-analysis.

Genetic model	Test	t	95% CI	*P*
A vs. G	Begg’s test			1.00
	Egger’s test	1.73	−35.62, 46.85	0.334
AA + GA vs. GG	Begg’s test			1.000
	Egger’s test	0.24	−62.37, 64.73	0.852
AA vs. GA + GG	Begg’s test			0.296
	Egger’s test	54.02	6.03, 9.74	0.012
AA vs. GG	Begg’s test			1.000
	Egger’s test	1.43	−38.81, 48.65	0.389
