# Supplementary material for: Possible association of CCDC62 rs12817488 polymorphism and Parkinson’s disease risk in Chinese population: a meta-analysis
Source: Sci Rep. 2016 Apr 1;6:23991. doi: 10.1038/srep23991 (PMC4817521; doi:10.1038/srep23991)
Supplement: Supplementary Information [file srep23991-s1.pdf]

Possible association of CCDC62 rs12817488 polymorphism and Parkinson's disease risk in Chinese population: a meta-analysis

Yanjun Lu[1], Lu Tan[2], Na Shen[1], Jing Peng[1], Chunyu Wang[1], Yaowu Zhu[1], Xiong Wang[1#]

1. Department of Laboratory Medicine, Tongji Hospital, Tongji Medical College, Huazhong University of Science and Technology, Wuhan 430030, China.
2. Key Laboratory for Molecular Diagnosis of Hubei Province, The Central Hospital of Wuhan, Tongji Medical College, Huazhong University of Science and Technology, Wuhan, Hubei 430014, China.

#Corresponding author: Xiong Wang, Email: wangxiong@tjh.tjmu.edu.cn

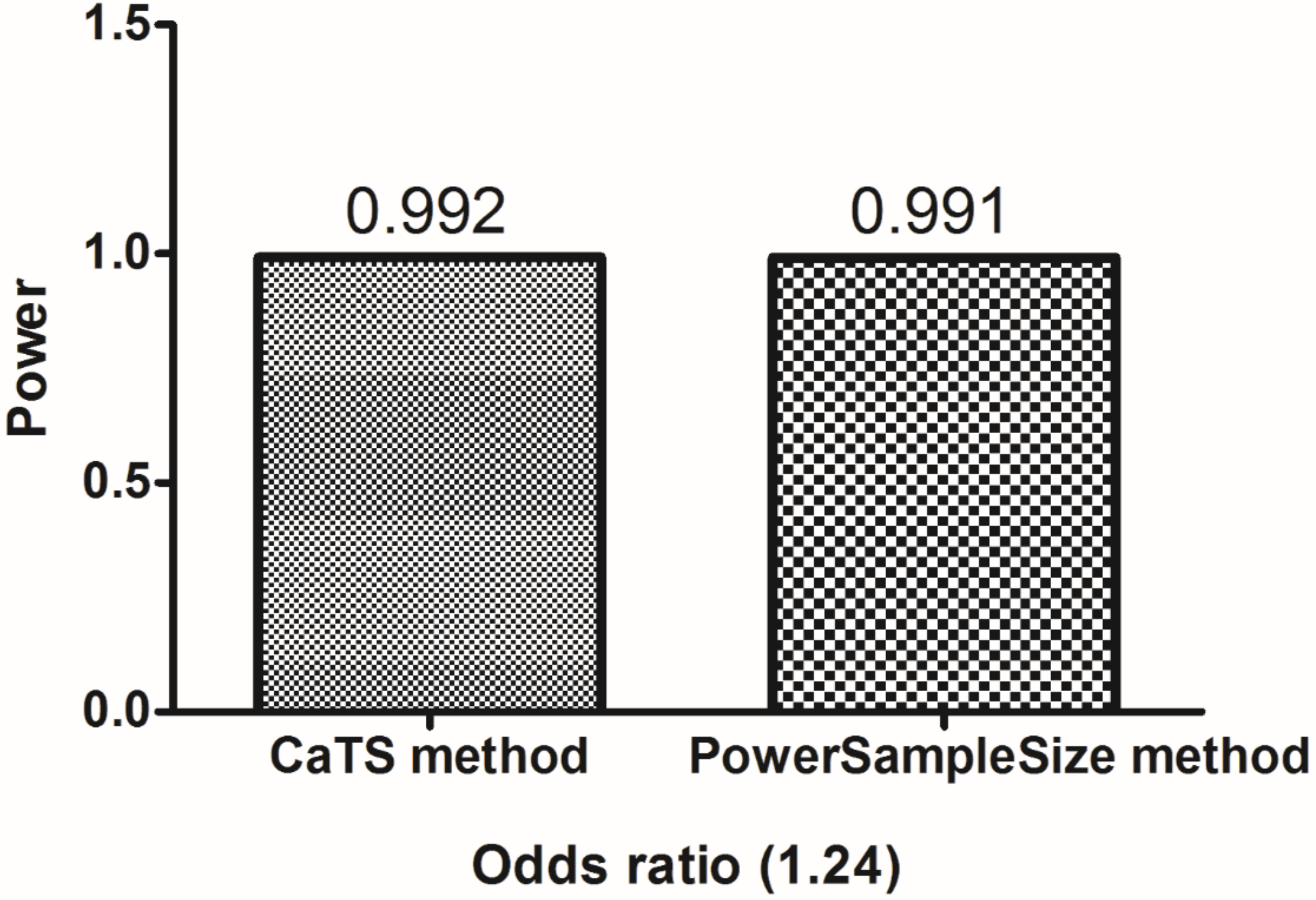

1.5
1.0
0.5
0.0
Power
0.992
0.991
CaTS method
PowerSampleSize method
Odds ratio (1.24)
